# Patients’ usability of seven most used dry-powder inhalers in COPD

**DOI:** 10.1186/s40248-019-0192-5

**Published:** 2019-09-13

**Authors:** Roberto W. Dal Negro, Paola Turco, Massimiliano Povero

**Affiliations:** 1 National Centre for Respiratory Pharmacoeconomics and Pharmacoepidemiology, Verona, Italy; 2 Research & Clinical Governance, Verona, Italy; 3 AdRes Health Economics and Outcome Research, Torino, Italy

**Keywords:** Dry powder inhalers, COPD, Global usability score, Inhalation therapy, Usability

## Abstract

**Introduction:**

Inhalation devices affect both the effectiveness and the therapeutic outcomes in persistent airway obstruction, and the effects are largely independent of the drug(s) assumed. Usability is a complex and comprehensive indicator of inhalation devices’ performance. The Global Usability Score (GUS) Questionnaire is an investigational tool designed to assess objectively the patients’-related and unrelated domains of devices’ usability.

**Methods:**

The GUS questionnaire was administered to all consecutive COPD patients referring for three months to the Lung Unit of CEMS Specialist Centre (Verona, Italy). The usability of seven Dry Powder Inhalers (DPIs) indicated as appropriate in COPD was tested and compared: Breezhaler, Diskus, Ellipta, Genuair, Nexthaler, Spiromax, and Turbohaler. Patients were divided in two groups, checked separately, according to their DPIs previous experience. A Bayesian Indirect Comparison (IC) model was built to assess “global usability” ranking.

**Results:**

A total of 103 patients were investigated: 74 patients already instructed in DPI use and 29 naive to DPIs. IC analysis proved Ellipta as the device characterized by the highest usability, while Breezhaler the device with the lowest usability in both groups of COPD patients (both with probability > 90%). Moreover, Turbohaler ranked second according to the Bayesian pooling, followed by Diskus, Spiromax, Nexthaler, and Genuair in patients already instructed in DPI use, while the ranking order was not as much well defined in naïve patients, likely due to their too small sample.

**Conclusions:**

Usability is a multifaceted indicator that contributes to assess the factual DPIs’ convenience in real life. DPIs are characterized by different levels of real-life usability, which can be checked, compared and ranked by means of the GUS score.

**Electronic supplementary material:**

The online version of this article (10.1186/s40248-019-0192-5) contains supplementary material, which is available to authorized users.

## Introduction

Effective actions oriented to increasing both the awareness and the empowerment of patients suffering from Chronic Obstructive Pulmonary Disease (COPD) who need long-term inhalation therapy raised powerfully during the last decade, being the concept of adherence to treatment, together to that of personalized therapy, strongly supported [1].

Due to the continuous growing in the number of inhalation devices of different technologies, increasing evidence proved that patients are unable to use all inhalers equally well, and the training with inhalers should have consequently been regarded as a priority challenge [1, 2]. On the other hand, a substantial bulk of data showed that inhalers represent a critical factor *per sé* as they may affect the therapeutic outcomes substantially, even independently of the molecules used [3–6].

Several aspects of patients’ adherence to inhalation treatments had been extensively investigated. In particular, the determinants of patients’ insufficient adherence were mostly related to those of patients’ preference, or acceptance, or satisfaction by the majority of Authors [7–13]. Patients’ subjectivity and empowerment were consequently highly valued from this point of view, even if the correspondence between patients’ beliefs, DPIs’ performances, and their effective usability had been investigated with specific instruments only in few studies in real-life [1, 14–19].

The Global Usability Score (GUS) Questionnaire is a comprehensive and anonymous operational instrument specifically developed for assessing and compare objectively the global usability of inhalation devices [20]. The GUS questionnaire was preferred to other instruments available in the literature [11, 16, 17, 19] because it allows the assessment of a much wider range of domains, thus resulting in a more comprehensive usable score.

### Aim

The aim of the study was to assess and compare the usability of the seven most used Dry Powder Inhalers (DPIs) in DPI naïve and in educated COPD patients.

## Methods

In order to investigate the usability of different DPIs, the GUS questionnaire [20] was administered to all consecutive COPD patients referring to the CEMS Specialist Centre (Verona-Italy) during the trimester October–December 2017. The GUS questionnaire is an anonymous operational instrument specifically developed for assessing and comparing objectively the usability of different inhalation devices simultaneously. This questionnaire was chosen because it allows the investigation of a wider range of factors affecting usability, and takes in the right value the nurse’s controlled assessment of patients’ inhalation procedures (see the Additional file 1). In order to avoid any influence of the subjects responses, the values corresponding to subjects’ responses to each item of the questionnaire (see Additional file 1) were obviously not reported in the version of Questionnaire distributed to subjects (otherwise their responses would be influenced), being the final GUS calculated by the steering committee only once the questionnaire had been filled definitively.

**Fig. 1:**
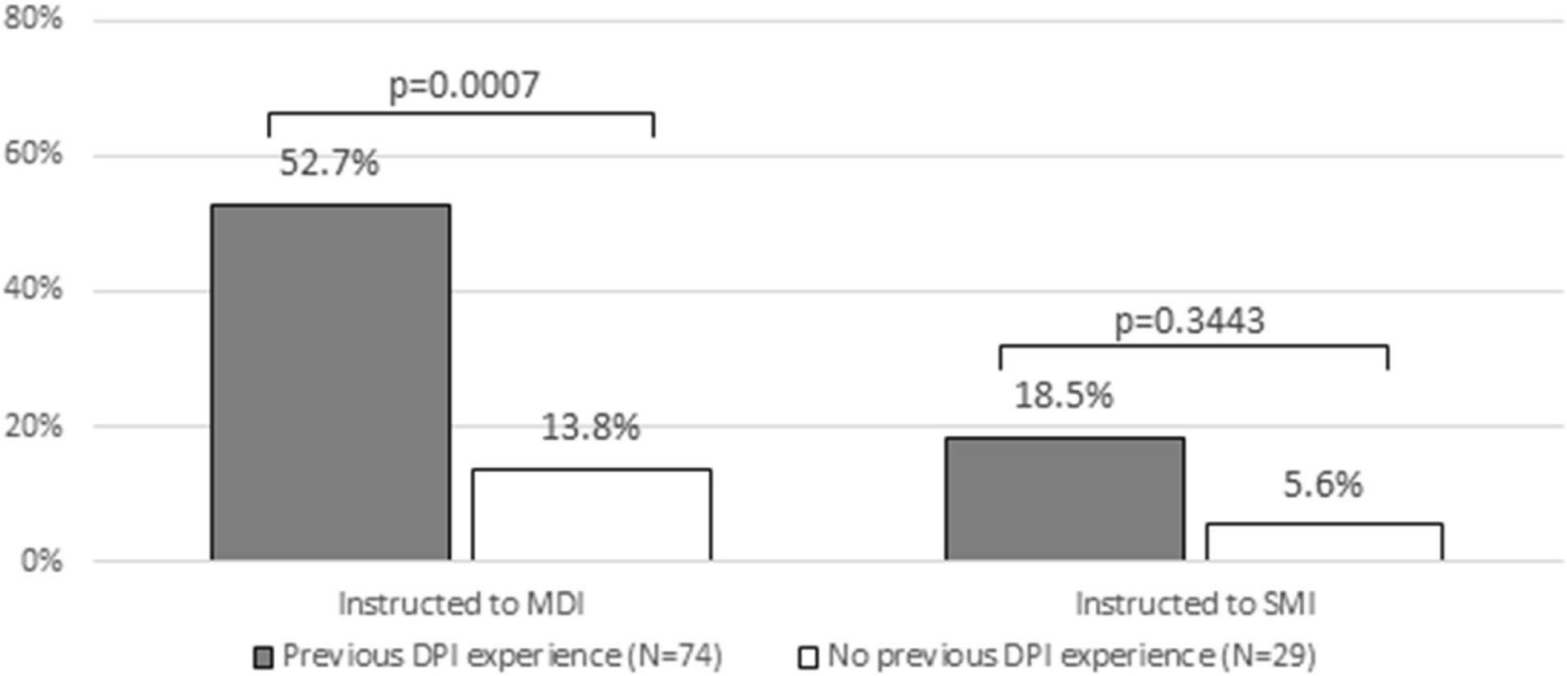
Previous experience with MDI and SMI: comparison between DPI experienced patients and DPI naïve patients. DPI: dry-powder inhalers; MDI: metered dose inhalers; SMI: soft mist inhalers

DPI experienced were enrolled together to naïve subjects just in order to investigate whether or not DPIs’ usability can be affected by previous subjects’ experiences with DPIs. Obviously, naïve subjects, even though having experienced inhalation therapies in their past, had never experienced any DPI previously.

The seven most used DPIs officially indicated in COPD were checked: Breezhaler, Diskus, Ellipta, Genuair, Nexthaler, Spiromax, and Turbohaler. Each patient was asked to evaluate up to four devices (randomly grouped) in the same session.

Two nurses, interchangeable because equally expert and motivated in educational programs, and familiar with the technical and the psychological aspects of the GUS Questionnaire, were specifically dedicated to patients’ interviews, and to supervise, check, assess, and validate all patients’ procedures for inhalation. The study consisted in four different steps:

- Step 1 - The attending nurse investigated the basic knowledge of each patient in DPI use.
- Step 2 - The nurse displayed the correct functioning of each DPI to each patient (already instructed or naïve) in random order. All explanations were provided according to a fixed sequence of sentences, being their duration previously standardized. Patients were then requested to declare their preference at glance and to specify the reason for their choice. All information collected during this phase were reported in the # 1 box of the Assessing Track (AT) section of the questionnaire (see Additional file 1).
- Step 3 - Patients were then requested to prepare the actuation of each device by themselves, while the nurse was monitoring and assessing the patients’ technicality. Their critical issues had to be valued; the number of attempts needed for actuating the device properly counted, and the overall time spent measured. Data on patients’ beliefs, the reasons of their choices, and quantitative data of nurse’s direct measurements were reported in boxes # 2–4 of the GUS questionnaire (see Additional file 1).
- Step 4 - Finally, data from other ten closed questions related to further subjects’ personal beliefs attaining to the acceptance and the preference of each device, were collected and reported in box # 5 (see Additional file 1).

At the end of each box of the Assessing Track, a sub-score is calculated. At the end of the whole questionnaire, the final Global Usability Score is easily obtained by summing-up all the sub-scores calculated for each DPI. The GUS final score ranges 0–50 points; higher the value of the score for each DPI, higher the corresponding usability will be.

### Ethics

The study was approved by the Ethical and Scientific Commission of the National Centre for Respiratory Pharmacoeconomics and Pharmacoepidemiology during the session of January 4^th^, 2016 (# RD 011/G01/16).

Only data collected from patients who gave their informed consent to the investigation and to the possible use of information for scientific purposes were used for the present study.

### Statistics

Patients’ characteristics were summarized as percentage for dichotomous and categorical variables, or as mean (± standard deviation) for continuous data. Difference in baseline characteristics among patients who tested the different sequences of devices were tested by means of chi-squared test for dichotomous/categorical or by ANOVA test for continuous variables.

All pairwise comparisons between the seven DPIs were merged using a Bayesian Indirect Comparison (IC) model [21]. Generally, IC models are used for pooling quantitative results from multiple studies and for assessing the effect between two or more treatments; in our context, “multiple studies” meant groups 1, 2, 3, and “treatments” means devices. This approach is particularly advantageous because all devices under comparison are incorporated into a single model even if they are not compared in the same questionnaire. Furthermore, the Bayesian technique enables rank ordering of each device (i.e. the probability associated to each one being the 1^st^, 2^nd^, 3^rd^, … best device) hence it results in a “global usability” ranking. Both fixed-effect (FE) and random-effect (RE) model were run. A FE analysis assumes that each individual generates an estimate of the same effect *d* (the preference of one device rather than another, in this case), subjected to sampling error; in a RE model, each individual *i* provides an estimate of the device effect *δ*_*i*_, which is not equal but similar to the real effect that is each effect *δ*_*i*_ comes from a normal distribution with mean *d* and variance σ^2^ representing the variability between respondents. The relative goodness of fit of the models was assessed by using the Deviance Information Criterion (DIC). Both FE and RE models were developed and the one associated to the lowest DIC was selected [22], with a difference of at least three points in DIC [23]. The model with the smallest DIC is the model with the best compromise between adequacy and complexity. Estimated GUS score for each device was presented as mean and 95% credibility interval (CrI).

Statistical analyses were performed with R statistical software version 3.1.2 [24], a *p* lower than 0.05 is considered to indicate evidence of differences in the evaluated variables. The Bayesian IC model was developed by using the software package WinBUGS 1.4.3 [25].

## Results

A total of 103 consecutive COPD patients were enrolled during ambulatory visits. The main characteristics of the whole sample were: males =61.6%; mean age = 66.8 years±8.2 sd; mean BODE Index = 4.3 ± 2.7sd; mean FEV_1_ (L) = 1.48 L ± 0.51 sd; mean FEV_1_% pred. = 56.3% ± 16.2sd; mean % reversibility from baseline = 5.2 ± 5,2sd; active smokers were 23.3% (*n* = 24), while former smokers 64.1% (*n* = 66), and never smokers 12.6% (*n* = 13). Their presumed mean COPD duration was 9.8 years±7.9sd and their mean value of Charlson Comorbidity Index was 3.2 ± 1.7sd. Their mean rate of COPD exacerbations was 1.6 ± 1.4sd and the mean rate of hospital admissions was 0.6 ± 0.7 during the previous twelve months.

A proportion of 28% (*n* = 29) were subjects naïve to DPIs (such as, they had never received a DPI prescription previously) while 72% of patients (*n* = 74) had already experienced DPIs (Fig. 1). In particular, the latter subgroup of subjects were prescribed and experienced almost all DPIs available on the market during the last decade for different periods.

Patients were divided in 3 groups, evenly distributed according the DPIs tested: Group 1, 36 patients who tested Breezhaler, Spiromax, Nexthaler, and Ellipta; Group 2, 37 patients who tested Breezhaler, Spiromax, Diskus, and Turbohaler, and Group 3, 30 patients who tested Breezhaler and Genuair. Baseline patients’ characteristics are reported in Table 1. Their age, sex and education distribution were similar among the three groups; only their geographical distribution was different in patients previously experienced in DPIs use.

**Table 1:** General characteristics of patients who evaluated devices using the Global Usability Score questionnaire

	Instructed to DPI (*n* = 74)				Not instructed to DPI (*n* = 29)			
	Group 1	Group 2	Group 3	*p*	Group 1	Group 2	Group 3	*p*
N	27	28	19		9	9	11	
Mean age (SD)	68.6 (12.3)	69 (10.4)	67.6 (12.4)	0.9234	58.9 (9.0)	64.3 (13.4)	64.9 (11.2)	0.4578
Sex (male)	62.5%	64.3%	57.9%	0.9046	22.2%	33.3%	45.5%	0.5515
Country^a^				0.0002				0.9999
North	92.3%	39.3%	63.2%		66.7%	66.7%	63.6%	
Center	3.8%	17.9%	10.5%		33.3%	33.3%	27.3%	
South and Islands	3.8%	42.9%	26.35		0.0%	0.0%	9.1%	
Education^a^				0.1658				0.5683
Primary	13.0%	7.7%	10.5%		0.0%	11.1%	0.0	
Lower secondary	26.1%	57.7%	42,1%		44.4%	66.7%	57.9%	
Upper secondary	56.5%	30.8%	42,1%		44.4%	22.2%	31.6%	
Degree	4.3%	3.8%	5,3%		11.1%	0.0%	10.5%	

Group1 tested Breezhaler, Spiromax, Nexthaler, and Ellipta; Group 2 tested Breezhaler, Spiromax, Diskus, and Turbohaler; Group 3 tested Breezhaler and Genuair; *DPI* dry powder inhalers, *SD* standard deviation

^a^ Information about country and education are reported also for Group 3, even though available only a few patients who answered those questions

As expected, experienced patients were also more instructed in the use of metered dose inhalers (MDI) than naïve patients (52.7% vs 13.8%, delta = 38.9, 95% CI 19.6─58.2%, *p* = 0.0007); the same trend was also observed for those previously experienced in the use of soft mist inhalers (SMI), even if the difference did not reach the statistical significance in this case (18.5% vs 5.6%, delta = 12.9, 95% CI -5.6─31.5%, *p* = 0.3443).

**Fig. 2:**
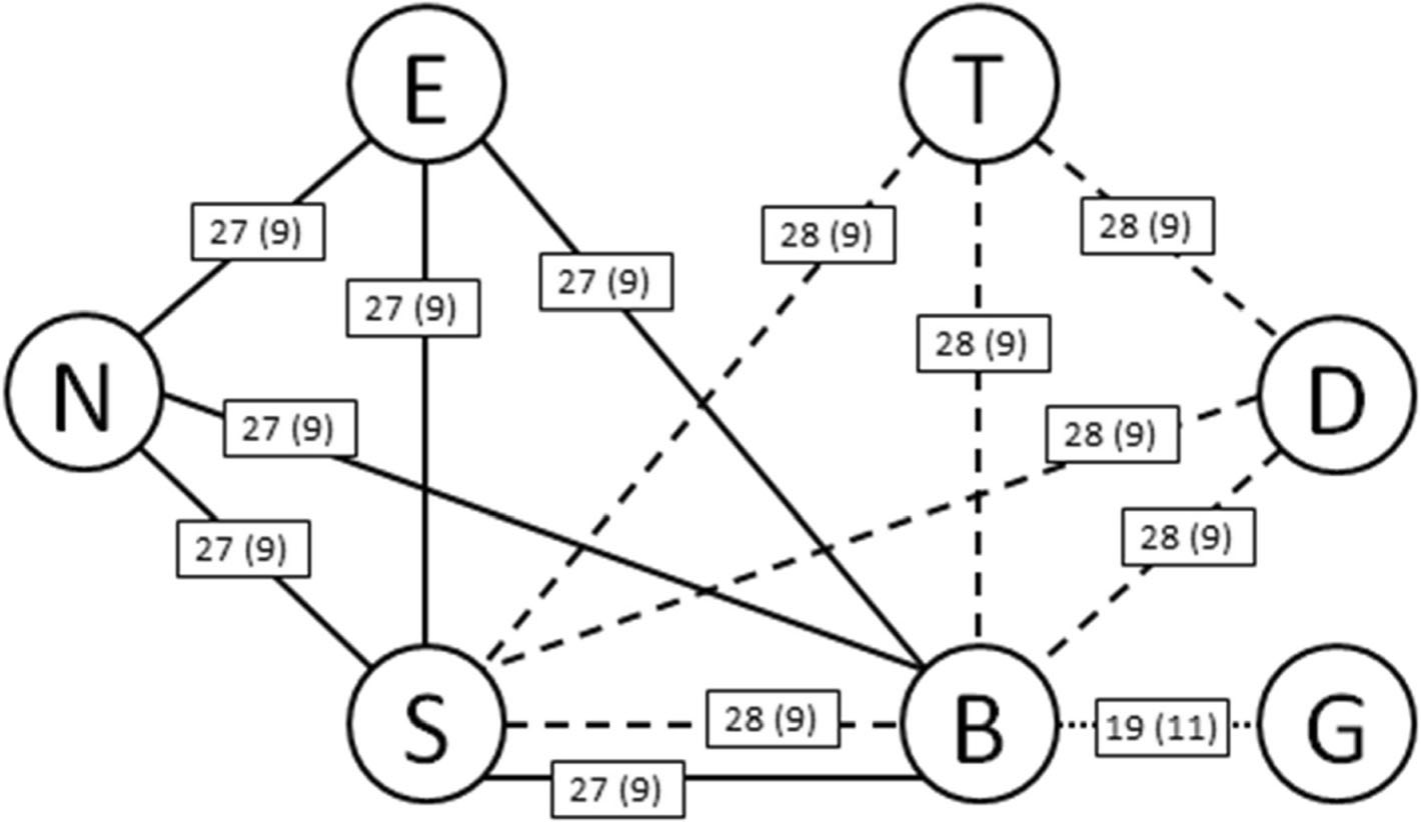
Evidence network based on patients’ comparisons in the three groups, every edge between two nodes is labelled with the number of patients that compared the devices represented with previous experience in the use of DPI (in brackets those without previous experience); solid lines represent comparison in group 1, dotted lines represent comparisons in group 2 and point line represents comparison between Breezhaler and Genuair in group 3 B: Breezhaler; D: Diskus; E: Ellipta; G: Genuair; N: Nexthaler; S: Spiromax; T: Turbohaler

Evidence network resulted from the 103 questionnaires is illustrated in Fig. 2. Ellipta, Nexthaler, and Breezhaler are directly compared only in group 1; Turbohaler, Diskus, and Spiromax are directly compared only in group 2; Breezhaler and Spiromax are directly compared both in group 1 and 2 while Genuair is compared with Breezhaler only in group 3.

In running our Bayesian analysis, the value of DIC was found to be more favorable for the FE model than the RE model, both for the DPI experienced population (DIC = 53.512 in FE model vs 53.921 in RE model) and for the DPI naïve population (DIC = 58.652 in FE model vs 58.702 in RE model). As explained in the statistics section, only the results generated by this model are presented since the model with the smallest DIC is the model with the best compromise between adequacy and complexity.

**Fig. 3:**
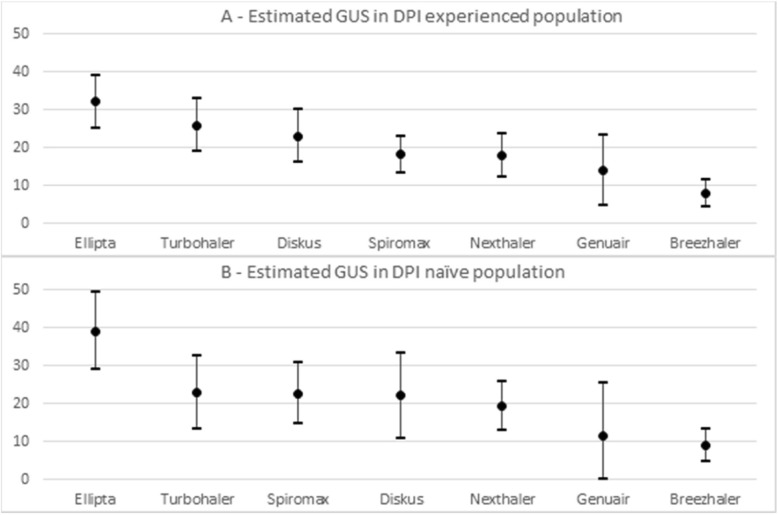
Ranking of mean GUS score (points) and 95% CrI (bars) resulting from Bayesian analysis in COPD patients originally already instructed to DPIs (**a**) and originally naive to DPIs (**b**). DPI: dry powder inhaler, CrI: credibility interval

The estimated GUS value for each device considered in the study are reported in Fig. 3. The usability of Ellipta and Breezhaler proved independent of the patient’s original level of previous instruction; furthermore, the two devices were characterized by the highest (Breezhaler) and the lowest (Ellipta) GUS score. However, only in the DPI experienced group (Fig. 3-a), the trend in usability proved quite linear from Ellipta (with the highest GUS) to Breezhaler (the lowest GUS). In the naïve group, GUS scores of Turbohaler, Spiromax, Diskus, and Nexthaler resulted very similar and also 95% CrI were very close (Fig. 3-b).

**Fig. 4:**
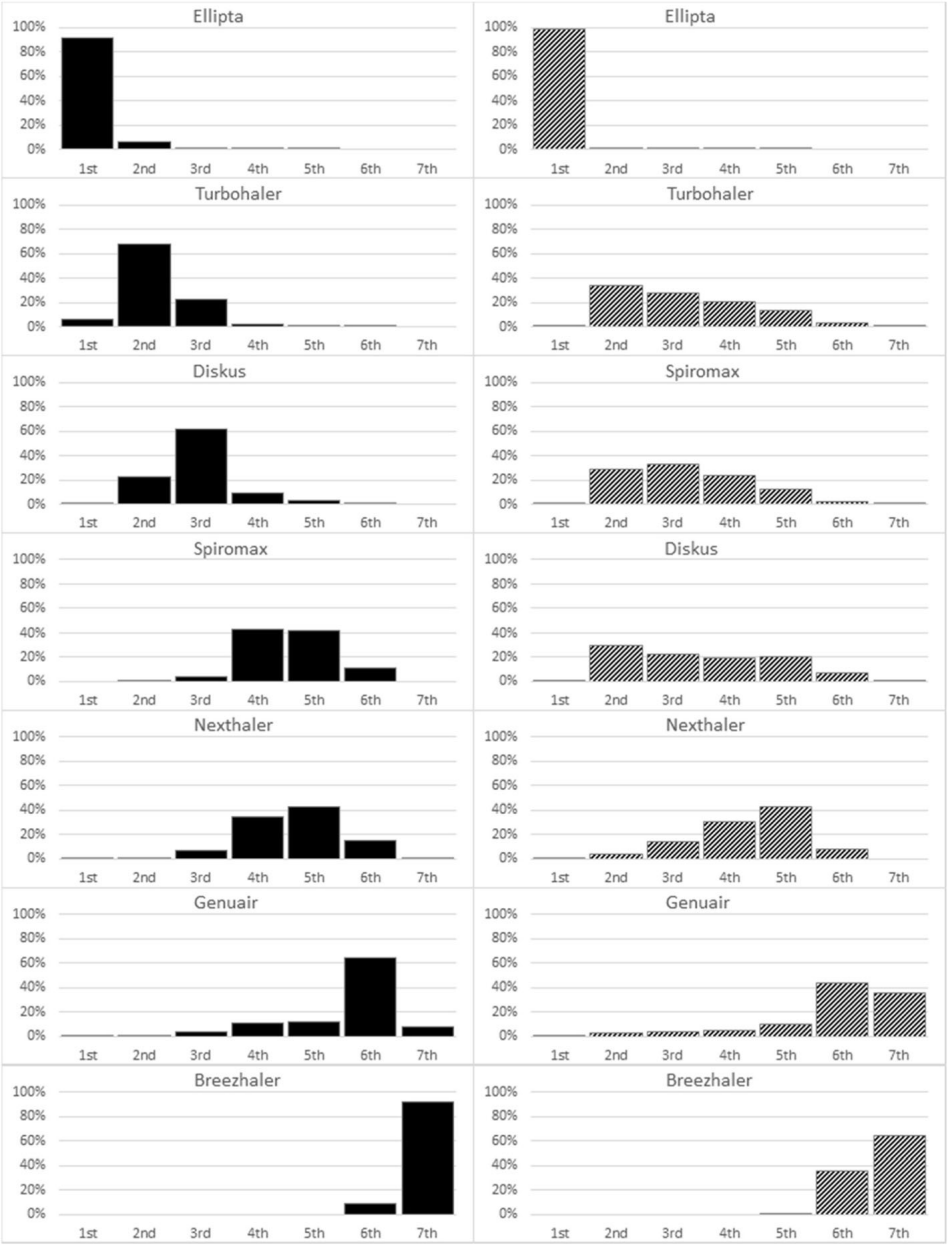
Histograms of rankings generated by the Bayesian pooling (patients instructed to DPIs on the left, not instructed on the right): the graphs reflect a total of 100,000 iterations and consist of as many histograms as the devices included in the analysis; in each panel, the histogram shows the percent distribution of the simulations across ranks 1 (greater GUS) through 7 (lower GUS) while the y-axis shows probability on a 0 to 1 scale

The histograms of rankings generated by the Bayesian pooling in originally instructed and in naïve COPD patients are reported in Fig. 4. The graphs reflect 100,000 iterations and consist of as many histograms as the devices included in the analysis. In each panel, each histogram shows the percent distribution of the simulations across ranks 1^st^ (the greatest GUS) to 7^th^ (the lowest GUS), while the y-axis shows the probability on a 0 to 1 scale. As anticipated by Fig. 3 for the DPI experienced COPD patients, the individual ranking of the 7 devices was straightforward: Ellipta 1^st^ (up to 2^nd^), Turbohaler 2^nd^ (1^st^ to 3^rd^), Diskus 3^rd^ (2^nd^ to 5^th^), Spiromax tied with Nexthaler (4^th^ to 6^th^), Genuair 6^th^ (4^th^ to 7^th^), and Breezhaler 7^th^ (6^th^ to 7^th^). In particular, Ellipta had more than 90% probability to be the first preferred device; Turbohaler had the highest probability to rank 2^nd^ (68%), but there was also a 23% to be 3rd. For the naïve population the ranking order did not prove as much definable: even if Ellipta has almost 100% of probability to be the first and Breezhaler 35 and 64% of probability to be the 6^th^ or the 7^th^, respectively; the ranking of the other five devices appeared evenly distributed between rank 2^nd^ and rank 6^th^. In particular, Turbohaler, Spiromax, and Diskus were be distinguishable between ranks 2 and 4.

## Discussion

The choice (and switching) of inhaler devices, in particular of DPIs, is a crucial issue in real-life studies since long ago [26]. Systemic reviews on randomized clinical trials did not confirm this evidence because mostly focusing the role of drugs used in the majority of cases. For this reason, patient-centered studies in community setting and/or in unselected samples of subjects had been encouraged in order to provide data much more fitting with real-life conditions and patients behavioral habits [2, 27].

Actually, even if the patients’ opinion has been progressively valued much more than in the past, the assessment of aspects which are highly independent of patients’ personal viewpoint (such as, the device engineering, the training track and the training costs) should further contribute to provide a much more comprehensive and objective picture of the so called “usability” of inhalation devices.

In real life, DPIs are highly prescribed in Italian COPD patients being their prescription usually independent of their known basic characteristics and technical differences, such as the different number of main actions required for their actuation (7 for Breezhaler, 4 for Ellipta and Turbohaler, and 3 for the remaining devices), and their intrinsic resistance, ranging from 0.017 kPa^0.5^ L/min to 0.039 kPa^0.5^ L/min [28, 29].

Actually, different DPIs confirmed to provide different performances in terms of patterns of lung deposition and inspiratory flow required for an adequate inhalation. On the other hand, both these factors which mainly attain to the intrinsic engineering of the devices [2, 26, 30], are equally able to affect clinical outcomes, being patients almost completely unaware of their relative value from this point of view [31]. These aspects are recently receiving increasing, even if still insufficient, attention by prescribers. In a multinational survey conducted among primary and secondary care physicians, more than 30% of them considered the device before considering the respiratory drug to prescribe. Moreover, the vast majority of UK health professionals (87%) affirmed to be concerned about possible problems arising from therapeutic prescriptions if the inhalation device is not specified, and 86% of physicians were convinced that DPIs are non-interchangeable and that their unmotivated substitution would have an adverse impact [32].

In several studies (even if mostly consisting of small samples of subjects), the criteria for DPIs preference have been usually related to factors which are strictly dependent of patients’ characteristics, beliefs, and subjectivity, such as: their age, visual acuity, hand strength, coordination, cognition, psychological profile, socio-economic status, and educational level. As a consequence, satisfaction, or intuitivity, or willingness to use, or preference “at glance”, or dexterity, or ease-of-use, or acceptance were from time to time the indices used for assessing and comparing different DPIs [14, 28, 33, 34].

In the last decade, the term “usability” appeared in the literature, but “usability” is ever more frequently used as a synonymous of “ease of use”, even if “usability”, *per sé*, encases a more complex and multifaceted concept, which depend of several determinants from different domains.

In our opinion, “Usability” should be regarded as a much more comprehensive parameter and its effective value results from the weighted mix of subjective (namely, intuitivity, satisfaction, willingness to use, preference “at glance”, dexterity, ease-of-use, acceptance, etc.) and objective (such as, those independent of patients’ convincement and beliefs, cost included) determinants, all contributing to balance the role of different factors affecting the overall DPIs performance and convenience in real life. To point out that the cost related to DPIs use should also be included among the decision criteria of usability, even if it occurred episodically and usually only related to the cost reimbursement [6, 35].

The existence of different domains affecting DPIs usability is clearly confirmed in the present investigation, which was founded on data collected by means of the multidimensional GUS Questionnaire. To pinpoint that also the patient-dependent criteria of choice (namely, preference and ease-of-use, etc.) were carefully assessed (before instruction) and compared (after instruction) by the expert nurses who provided specific scores in order to check and measure the efficacy of the training track with each DPI.

To emphasize that no DPI achieved the theoretical GUS top score of 50 points, thus confirming that the “ideal DPI” is not still available and that, even if well performing, all presently available DPIs are affected by some critical aspects.

Nevertheless, some relevant differences among the seven DPIs used in the present study clearly came out in terms of their usability and, consequently, of educational actions to deliver for boosting their proper utilization. In particular, Ellipta and Breezhaler proved equally independent of patient’s original level of instruction, even if they were characterized by dramatically different GUS scores (such as, the highest and the lowest, respectively). Actually, this dramatic difference in GUS value may be likely suggested as due to the fact that, differently from all other six DPIs which are multi-dose inhalers, Breezhaler is a single-dose inhaler characterized by a very low intrinsic resistance which requires a very high subject’s inspiratory flow for achieving an effective drug inhalation, and several manoeuvres for its proper actution.

All the other remaining five devices seem to be influenced by a previous DPIs’ experience and their ranking proves very clear according to the GUS score: Turbohaler shows almost 70% of probability to be the 2^nd^; Diskus has 62% probability to be the 3^rd^; both Spiromax and Nexthaler have 80% probability to be the 4^th^, and Genuair has 65% probability to be the 6^th^.

On the other hand, the corresponding trend assessed in naïve COPD subjects does not result as much clear since Turbohaler, Spiromax, Diskus, and Nexthaler showed a probability of 12–34% to rank as the 2^nd^ up to the 5^th^ most usable device, followed by Genuair ranking 6^th^ or 7^th^ (with a probability of 44 and 35%, respectively), and finally Breezhaler ranking 7^th^ with a probability of 64%. This less defined trend was likely due to the small number of naïve patients evaluated (9 patients in both group 1 and 2, and 11 in group 3) which contributes to increase uncertainty in the analysis; in fact, the 95% CrI presented in Fig. 3-b are larger than those presented in Fig. 3-a.

Moreover, Ellipta, Spiromax, Turbohaler, and Diskus were the DPIs needing the lowest time to spend for achieving the patient’s autonomy and the quickest to learn. Once again, even if the expert nursing of inhalation procedures represents a crucial aspect with all DPIs [8, 29, 36], a careful educational training is mandatory with some DPIs, which are characterized by lower usability scores.

This evidence is further confirming and stressing the original concept that subjects are unable to use all inhalers equally well [1, 2], and that usability consists in and depends of a much more complex set of determinants than simple patients’ perceptions. In particular, each single domain is not able to assess *per sé* the usability of a DPI, differently from the GUS score, which is able to provide the overall and objective measure of all domains contributing to each DPI usability. Actually, DPIs can be ranked more effectively in terms of their usability when all the components provided by the GUS Questionnaire are weighted together, such as the subjective and objective components of comparison and ranking.

In other words, the GUS score proved able to limit substantially the role of patient-dependent factors of choice, thus leading to a much more reliable assessment of each DPI usability in real life. These results are emphasized by those of the Bayesian model of comparison that confirmed the ranking of the seven different DPIs provided by the GUS score.

The present investigation has some limitations. The present study is a monocentric survey, even if patients participating were from several Italian regions. Geographical distribution was uneven in patients previously experienced in DPIs use: nonetheless, we believe that this does not represent a relevant confounding factor since the national attitude had never been associated to significant regional differences in DPIs’ prescription. Moreover, the original whole sample had to be divided in sub-groups because the GUS Questionnaire consents the comparison of only four devices simultaneously. On the other hand, the simultaneous comparison of more than four DPIs per patient would create severe problems in terms of suitability of their response to the questionnaire. Finally, independently of the strict control of both the quality and duration of the nurses’ explanation of each DPI, it was anyway possible that minimal differences would occur in transferring the messages, even if the high predictivity of GUS score tends to exclude the occurrence of substantial biases from this point of view.

However, a point of strength is that, as the study attains to COPD patients, only the seven mostly used DPIs officially indicated as appropriate for COPD management were considered and compared by means of a strict statistical procedure.

## Conclusions

Usability is a multifaceted indicator, which greatly contributes to the definition of DPIs convenience and choice. Usability should not merely mirror the patient’s belief (such as, a synonymous of ease-of-use or intuitivity), but it should be regarded as the documented and assessed patient’s skill in using inhalation devices effectively, to be measured and/or compared by means of a comprehensive and global score, namely the GUS score. DPIs confirms as characterized by different levels of usability. The GUS score, just because not merely linked to patients’ personal at glance and aesthetic beliefs, contributes to the suitable ranking of DPIs in terms of their real-life performance and convenience.

## Additional file

Additional file 1: The Global Usability Score Questionnaire. (DOCX 33 kb)

## Data Availability

Availability on written request to authors.

## Abbreviations

COPD: Chronic Obstructive Pulmonary Disease
CrI: Credibility interval
DPIs: Dry Powder Inhalers
FE: Fixed-effect
GUS: Global Usability Score
IC: Indirect Comparison
RE: Random-effect
