# Correction to: Patients’ usability of seven most used dry-powder inhalers in COPD

**DOI:** 10.1186/s40248-019-0204-5

**Published:** 2019-11-26

**Authors:** Roberto W. Dal Negro, Paola Turco, Massimiliano Povero

**Affiliations:** 1 National Centre for Respiratory Pharmacoeconomics and Pharmacoepidemiology, Verona, Italy; 2 Research & Clinical Governance, Verona, Italy; 3 AdRes Health Economics and Outcome Research, Torino, Italy

**Correction to: Multidiscip Respir Med (2019) 14:30**

**https://doi.org/10.1186/s40248-019-0192-5**

After publication of the Original research article [1] it was brought to our attention that the sentence at the bottom of the section entitled Discussion (pag 6 of 9, just below Fig. 4) must be corrected as follows:

“In real life, DPIs are highly prescribed in Italian COPD patients being their prescription usually independent of their known basic characteristics and technical differences, such as the different number of main actions required for their actuation (7 for Breezhaler, 4 for Turbohaler, and 3 for the remaining devices),” and their intrinsic resistance, ranging from 0.017 kPa0.5 L/min to 0.039 kPa0 .5 L/min [28, 29].
