# Supplementary material for: Patients’ usability of seven most used dry-powder inhalers in COPD
Source: Multidiscip Respir Med. 2019 Sep 13;14:30. doi: 10.1186/s40248-019-0192-5 (PMC6743127; doi:10.1186/s40248-019-0192-5)
Supplement: Supplementary file 1 — The Global Usability Score Questionnaire. (DOCX 33 kb) [file 40248_2019_192_MOESM1_ESM.docx]

**Additional file 1**

**The Global Usability Score Questionnaire**

## (Check with an X, or describe your answer) n.: ………

**INTRODUCTORY SECTION**

- Previous instructions to the DPI use? Yes No if YES, from whom? ...................

(Dry Powder Inhalers)

- Previous instructions to the MDI use? Yes No if YES, from whom? ...................

(Metered Dose Inhalers)

- Previous instructions to the SMI use? Yes No if YES, from whom ? ..................

(Soft Mist Inhalers)

**THE ASSESSING TRACK**

**1 - To the Nurse: please report the duration of your explanation (in sec.): A .... B .... C .... D .....**

**Questions to the patient, after the nurse’s explanation (only one exclusive choice):**

**1.a** Which device did you prefer “at glance” ***(1 point)* A B C D**

**1.b** Which device did you perceive as the easiest to use ? ***(1 point)*** **A B C**  **D**

**1.c** Which device did you perceive as the most difficult to use ? ***(0 point)*** **A B C**  **D**

**Sub-Score: ….. ….. ….. …..**

**1.c.1** why ?: ………………………………..……………………

1. **Questions to the Patient and to the Nurse, after the Patient’s practicing:**

**2.a** Grade devices by difficulties you encountered in their use (by increasing order):

***(The Patient’s Opinion : 1=4 points ; 2=3 points ; 3=1 point ; 4=0 points)***

**1^st^ …… 2^nd^ …… 3^rd^ …… 4^th^ ……..**

**Sub-Score : …… …… …… …….**

**2.b** Please, report your most difficult step in actuating each device:

*(****The*** ***Patient’s Opinion****)*

**A …………………… B…………………… C………………….. D……………………..**

**2.c** Please, grade devices by difficulties the patient encountered (by increasing order):

*(****Nurse’s Assessment : 1=4 points ; 2=3points ; 3=1points ; 4=0 points****)*

**1^st^ …… 2^nd^ …… 3^rd^ …… 4^th^ ……..**

**Sub-Score : …… …… …… …….**

**2.d** Please, report the most difficult patient’s actuation step with each device::

***(The Nurse’s Assessment****)*

**A …………………… B…………………… C………………….. D……………………..**

1. **Please, report the overall n. attempts for the 1^st^ proper actuation with each device:**

***(The Nurse’s Assessment) (score: 1= 5 points; 2= 3 points; 3= 1 point; >3 = 0 points)***

**A …… B …… C …… D……..**

**Sub-Score:** -------- -------- ------- --------

1. **Total time spent for the 1^st^ proper actuation with each device (in sec.):**

***(The Nurse’s Assessment)***

***(score: <120”= 5 points; ≥120” <180”= 3 points; ≥180” < 360”= 1 point; ≥360”= 0 point)***

**A …… B …… C …… D ……..**

**Sub-Score** -------- -------- ------- -------

1. **Please indicate which device you prefer (only one exclusive choice/item) in terms of :**

***(The value of each Patient’s item: items a - d = 1 point; items e - f = 3 points; g - j = 5 points)***

**Device ------------------------------------**

1. **Shape *(1 point)* A B C D**
2. **Size  *(1 point)* A B C D**
3. **Mouthpiece *(1 point)* A B C D**
4. **Hygiene *(1 point)* A B C D**
5. **Presence of a dose counter *(3 points)* A B C D**
6. **Ease of gripping *(3 points)* A B C D**
7. **N. manoeuvres for actuation *(5 points)* A B C D**
8. **Ease of use *(5 points)* A B C D**
9. **Perception of inhaled dose *(5 points)* A B C D**

1. **Presence of a trigger valve *(5 points)* A B C D**

**-------- -------- ------- -------**

**Sub-Score …….. ..….. …… …….**

**Please, sum the items’ 1;2;3;4, and 5 sub-scores:**

**The Global Score (*range 0-50*) …..…. …….. …….. .…….**

**Age:** ………… **Gender:** M F **Region of living**: ............................................

**Education**: 0 = none; 1 = elementary; 2 = middle; 3 = high; 4 = degree

**Thank you for your valuable contribution**
